# A Digital Twin Strategy to Predict Thrombotic Recurrence in Antiphospholipid Syndrome Patients Treated with Direct Oral Anticoagulants vs. Vitamin K Antagonists Using Data from Real-World Populations

**DOI:** 10.3390/jcm14165716

**Published:** 2025-08-12

**Authors:** Miguel Ángel Casado-Suela, Juan Torres-Macho, Aida Izquierdo-Martínez, Cristina Lucía Ancos-Aracil, Luis Ferreira-Burguillos, Elena Madroñal-Cerezo, Tamar Talaván-Zañón, Adela Castañeda-Mata, Luis Escobar-Curbelo, Ana Martínez de la Casa-Muñoz, Eva Ruiz-Navío, Ana Bustamante-Fermosel, Anabel Franco-Moreno

**Affiliations:** 1 Department of Internal Medicine, Hospital Universitario Infanta Leonor, 28031 Madrid, Spain; xelele54@gmail.com (M.Á.C.-S.); juan.torresm@salud.madrid.org (J.T.-M.); abustamantef@salud.madrid.org (A.B.-F.); 2 Department of Medicine, Universidad Complutense de Madrid, 28040 Madrid, Spain; 3 Department of Internal Medicine, Hospital Universitario de Fuenlabrada, 28942 Madrid, Spain; aida.izquierdo@salud.madrid.org (A.I.-M.); cristina.ancos@salud.madrid.org (C.L.A.-A.); lferreira@salud.madrid.org (L.F.-B.); elena.madronal@salud.madrid.org (E.M.-C.); 4 Venous Thromboembolism Unit, Hospital Universitario de Fuenlabrada, 28942 Madrid, Spain; 5 Clinical Analysis Laboratory, Hospital Universitario Infanta Leonor, 28031 Madrid, Spain; tamar.talavanz@salud.madrid.org; 6 Clinical Analysis Laboratory, Hospital Universitario de Fuenlabrada, 28942 Madrid, Spain; adela.castaneda@salud.madrid.org; 7 Artificial Intelligence, 11012 Cádiz, Spain; drescobar@gmail.com; 8 Hospital Universitario Gregorio Marañón, 28007 Madrid, Spain; amdelacasa@hotmail.com; 9 Hospital Universitario Infanta Leonor, 28031 Madrid, Spain; eva.ruiz@salud.madrid.org; 10 Venous Thromboembolism Unit, Hospital Universitario Infanta Leonor, 28031 Madrid, Spain

**Keywords:** antiphospholipid syndrome, digital twin, direct oral anticoagulants, machine learning, precision medicine, real-world data, thrombosis recurrence, vitamin k antagonists

## Abstract

**Background/Objectives:** The role of direct oral anticoagulants (DOACs) vs. vitamin K antagonists (VKAs) in preventing recurrent thrombosis in patients with antiphospholipid syndrome (APS) remains uncertain. Using real-world data, we aimed to evaluate the effectiveness and internal validity of a digital twin (DT) approach for modeling thrombotic recurrence risk in APS patients treated with DOACs or VKAs. **Methods:** We conducted a multicenter observational study that included thrombotic APS patients treated with DOACs or VKAs. Clinical data were used to generate DT via conditional generative adversarial networks (CGANs), incorporating a directed acyclic graph (DAG) to preserve causal relationships. Validation metrics included absolute standardized mean differences (ASMD), mean ASMD (MASMD), and Spearman correlation matrices to assess structural fidelity. Treatment effects were estimated in a CGAN-conditioned cohort matched on key covariates. **Results:** Eighty-nine thrombotic APS patients were included: 70 (78.7%) received VKAs and 19 (21.3%) received DOACs. Thrombotic recurrences occurred in 5 DOAC patients (26.3%) and 17 AVK patients (24.3%). The CGAN-generated synthetic cohort closely mirrored the original data (MASMD = 0.073 ± 0.041), with 85.4% of pairwise correlations differing by <0.1 in absolute value. In the conditioned DT cohort, predicted recurrence was 24.2% for DOACs and 19.9% for VKAs. Recurrence risk increased with antibody burden, reaching 41.3% in triple-positive patients and 46.8% in those with index arterial thrombosis treated with DOACs. **Conclusions:** DT technology accurately replicated the clinical structure of APS patients, supporting its application for simulating counterfactual scenarios and estimating individualized treatment effects.

## 1. Introduction

Antiphospholipid syndrome (APS) is the most frequently acquired thrombophilia related to autoimmune disorders. It is characterized by the occurrence of arterial and/or venous thrombotic events at macro- and microvascular circulation, as well as pregnancy complications, all in the presence of antiphospholipid antibodies (aPLs). These antibodies include lupus anticoagulant (LA), anti-cardiolipin antibodies (aCLs), and anti-beta-2-glycoprotein I antibodies (anti-β2GPI). The American College of Rheumatology (ACR) and the European Alliance of Associations for Rheumatology (EULAR) have recently published updated classification criteria for APS. These criteria, published in late 2023, aim to improve APS patient identification [1].

APS is a paradigmatic thromboinflammatory syndrome. The pathogenesis of thrombotic APS is a complex interplay between inflammatory and coagulation pathways, with aPL antibodies playing a central role [2]. aPLs lead to the activation of vascular and immune cells, inhibit antithrombotic factors (e.g., protein C and plasminogen), and up-regulate procoagulant molecules, including tissue factor, factor V, and factor VIII, resulting in vascular thrombosis.

Thrombosis is the hallmark of APS. Stroke and venous thromboembolism (VTE) are the most frequent forms of presentation [3]. The mainstay of treatment for thrombotic APS is long-term anticoagulation. Evidence from long retrospective series shows a high rate of recurrent thrombosis in patients with APS who discontinued anticoagulant therapy [4,5].

When compared to vitamin K antagonists (VKAs), direct oral anticoagulants (DOACs) offer several potential advantages, such as fixed-dose prescriptions, no need for anticoagulant effect monitoring, reduced significant bleeding, especially intracranial, and fewer drug–drug and drug–food interactions. Four randomized controlled trials (RCTs) have evaluated the efficacy and safety of DOACs for secondary prevention in patients with thrombotic APS [6,7,8,9,10] (Table 1). The RAPS trial, comparing rivaroxaban 20 mg with warfarin in patients with APS and VTE, showed a lower rate of thrombin generation markers in the DOAC group. While the primary endpoint was not met, no recurrent thrombotic events occurred, and both treatments showed a similar safety profile regarding major bleeding [6]. The TRAPS study compared rivaroxaban 15 or 20 mg vs. warfarin in APS patients with triple-positive aPLs (LA, aCL, and anti-β2GPI). The significantly higher incidence of arterial thrombotic events, including strokes and myocardial infarctions, in the rivaroxaban group compared with the AVK group (12% vs. 0%) led to the early termination of the trial [7]. The trial conducted by Ordi-Ros et al. evaluated the efficacy and safety of rivaroxaban 20 or 15 mg compared with VKAs. After three years of follow-up, recurrent thrombosis occurred in 11.6% (11/95) patients in the DOAC group and 6.3% (3/95) in the VKA group. Notably, 9 of the 11 recurrences in the DOAC arm were strokes [8]. Similar results were observed in the ASTRO-APS trial comparing apixaban with VKAs, even after the protocol was modified by excluding patients with previous arterial thrombosis and duplicating the dose of apixaban from 2.5 to 5 mg twice daily [9]. A meta-analysis of the four RCTs reported an increased risk of arterial thrombosis (odds ratio [OR] 5.43), but not venous thrombosis (OR 1.20), of DOACs compared to VKAs [10]. In addition, triple aPL positivity was associated with recurrence (OR 5.65). Therefore, guidelines recommend VKAs as the mainstay of therapy to prevent thrombotic recurrence in patients with APS [11]. Nevertheless, these RCTs had small sample sizes. Although these studies have highlighted a potential increase in recurrent thrombotic events with DOACs compared to VKAs, particularly arterial thrombosis, they lack sufficient power to evaluate thrombotic outcomes in specific subgroups of populations. Therefore, the therapeutic balance between DOACs and VKAs remains unclear in this clinical setting.

Digital twin (DT) technology is emerging to transform healthcare research. DTs are advanced computational models replicating real-world patient populations by integrating clinical data with machine learning algorithms [12,13]. In the context of RCTs, DTs enable the creation of virtual cohorts that mirror the characteristics of specific subgroups, allowing for the evaluation of treatment effects under varying clinical scenarios. By conditioning DTs on specific covariates, it is possible to simulate outcomes in patient populations that may not have been adequately represented in the original RCTs. This approach enhances the external validity of trial findings and facilitates personalized risk stratification and predictive modeling in complex conditions [14].

APS is recognized as a rare disease by the Orphanet database (ORPHA 80 classification). Given the low incidence of thrombotic APS, generating solid evidence through other RCTs or prospective studies would require many years and large-scale recruitment efforts. In this context, DT-based approaches offer an innovative approach to more accurately assess the efficacy and safety of DOACs. We hypothesize that applying a DT model in patients with thrombotic APS, stratified by key clinical characteristics, will allow for a robust estimation of the treatment effects of DOACs compared to VKAs.

## 2. Materials and Methods

We conducted a multicenter, analytical, observational study following the STROBE recommendations for observational studies [15]. The study was approved by the Ethics Committee of the Hospital Universitario Clínico San Carlos (code 25/451-E) and carried out in accordance with the Declaration of Helsinki and Good Clinical Practice guidelines.

### 2.1. Patients and Study Setting

Patients with thrombotic APS treated with VKAs or DOACs from Hospital Universitario Infanta Leonor and Hospital Universitario de Fuenlabrada, both in Madrid, Spain, were included. The study period was between 1 January 2010 and 1 February 2024. The patients were classified as having APS based on the 2006 Sydney criteria [16]. In accordance with the 2006 Sydney criteria, we defined a positive result for aCL and anti-β2GPI antibodies as a titer ≥40 U/mL. LA was considered positive when confirmed by validated phospholipid-dependent clotting assays (e.g., dRVVT, aPTT) following the ISTH three-step procedure (screening, mixing, and confirmatory tests). Anticoagulant choice (DOAC or VKA) was determined by the responsible physician based on clinical criteria. All patients in the DOAC group received full-dose regimens according to approved indications, except for one patient on apixaban who was switched to a reduced dose after six months of treatment. The target INR range for VKA therapy was 2.0–3.0. No patients switched between anticoagulant groups during the follow-up period. Recurrence thrombotic events were confirmed by objective imaging. The index thrombotic event was defined as the first objectively documented arterial or venous thrombotic episode that led to the initiation of long-term anticoagulation and that fulfilled the clinical criterion of thrombosis required for the diagnosis of APS. Enrolled patients were located through the electronic registry of both centers’ venous thromboembolism (VTE) units. Data quality was regularly monitored.

### 2.2. Baseline Variables

Baseline variables included age, sex, weight, and the presence of coexisting clinical conditions, such as smoking, hypertension, diabetes mellitus, dyslipidemia, chronic cardiovascular or pulmonary disease, prior stroke, estrogen therapy, chronic kidney disease, concomitant autoimmune disorders, malignancy, another thrombophilia (including factor V Leiden mutation, prothrombin G20210A mutation, protein C or S deficiency, antithrombin deficiency), platelet count, hemoglobin, concomitant antiplatelet therapy, index thrombotic event, aPL profile, and follow-up duration. Additionally, major bleeding complications were recorded. According to the ISTH criteria, major bleeding is defined as overt bleeding requiring the transfusion of two or more units of blood or bleeding that is retroperitoneal, spinal, intracranial, intrathecal, intrapericardial, intraocular, or fatal [17].

### 2.3. Outcome

The outcome was thrombotic recurrence, defined as a new arterial or venous thrombotic event during follow-up. Only symptomatic thrombotic recurrences were assessed. All events were confirmed by objective diagnostic methods, including doppler ultrasound (Vivid S70N, GE Healthcare, Chicago, IL, USA) for deep vein thrombosis, computed tomography (CT) angiography (SOMATOM Definition AS, Siemens Healthineers, Erlangen, Germany) for pulmonary embolism and placental thrombosis, CT (SOMATOM Definition AS, Siemens Healthineers, Erlangen, Germany) and magnetic resonance imaging (Achieva 1.5T, Philips Healthcare, Best, The Netherlands) for stroke, and coronary angiography (Allura Xper FD20, Philips Healthcare, Best, The Netherlands) for myocardial infarction. Recurrence rates were compared between patients treated with DOACs and those receiving VKAs.

### 2.4. Digital Twin Creation

#### 2.4.1. Generative Modeling

DTs were generated using generative adversarial networks (GANs) and conditional GANs (CGANs) to replicate individual patient profiles with thrombotic APS. Patient-level data were harmonized to ensure consistency among variable definitions, handling missing values, and detecting outliers. Synthetic data generation was conditioned on clinically relevant covariates, such as treatment group, age, sex, index thrombotic event, aPL profile, and follow-up duration. These variables were selected based on their significance and availability across the dataset. Generated twins were validated against the original patient-level data by comparing the distributions of clinical outcomes and covariates to assess fidelity. The goal of validation was to ensure that the synthetic populations preserved statistical consistency and clinical plausibility concerning the real-world APS cohort. We used maximum mean discrepancy (MMD) and Kolmogorov–Smirnov tests to compare the marginal distributions of key continuous and categorical variables between real and synthetic datasets. In addition, to avoid clinically implausible synthetic profiles, the digital twin model included hard constraints and applied a post-generation filter. These strategies helped ensure that generated patients did not present physiologically impossible combinations.

#### 2.4.2. Variable Relationships and DAG Construction

**Figure 1:**
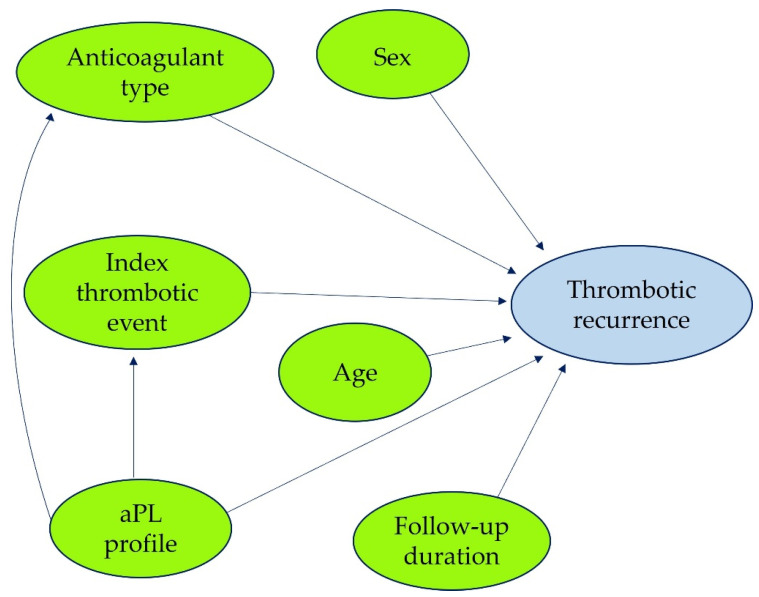
The DAG included the clinically relevant covariates and defined causal relationships between treatment allocation, baseline patient characteristics, and thrombotic recurrence in APS patients.

A directed acyclic graph (DAG) was incorporated into the CGAN training process to capture clinically meaningful relationships between covariates and to prevent spurious correlations. The DAG defined assumed causal relationships among selected variables and guided the dependency structure in synthetic data generation. From the harmonized dataset, the variables included in the DAG were treatment group, age, sex, index thrombotic event, aPL profile, follow-up duration, and thrombosis recurrence (Figure 1). Recurrence was included in the DAG as an outcome node to guide causal structure but was not used as a conditioning variable in CGAN training. The number of covariates in the DAG was deliberately limited to maintain interpretability and avoid overfitting, which aligns with recommendations from causal inference frameworks that emphasize parsimony and clinical relevance [18]. Expert clinicians defined the directionality of the edges based on clinical reasoning and available evidence. Variables with substantial missingness or limited clinical consistency, such as incomplete aPL titers or unstable International Normalized Ratio (INR) measurements, were excluded from DAG conditioning to ensure compatibility with the modeling framework. The final DAG included 8 directed edges. Integrating the DAG into the CGAN architecture enhanced the clinical plausibility of the generated digital twins by explicitly modeling dependencies among variables, thereby supporting more robust outcome simulations across a double-center, real-world APS population.

### 2.5. Model Evaluation (Digital Twins)

Following DT generation, logistic regression analysis was performed to assess the association between anticoagulation type (DOACs vs. VKAs) and recurrence. Mean absolute standardized mean differences (MASMDs) were computed for key variables to evaluate the covariate balance between the real and synthetic populations. A MASMD below 0.1 was considered indicative of adequate balance. To further explore internal data structure, Spearman correlation matrices were computed for key covariates, allowing for comparison of dependency patterns between the original and synthetic cohorts. Continuous variables were winsorized at the 2.5th and 97.5th percentiles to reduce the impact of extreme outliers prior to model training and analysis.

To assess the robustness and reproducibility of the synthetic data generation process, we created ten independent non-conditioned digital twin cohorts using the same CGAN configuration and training parameters. For each replica, validation metrics, including absolute standardized mean differences (ASMDs) and mean ASMD (MASMD), were calculated by comparing synthetic and real-world populations.

### 2.6. Sensitivity Analyses

Several sensitivity analyses were performed to assess the robustness of the findings. First, patients were stratified according to aPL profiles to evaluate the influence of serological phenotype. Second, a stratified analysis was conducted for index arterial and venous thrombotic events to examine the consistency of treatment effects within each subtype.

All analyses involving digital twin generation and validation were conducted using Python version 3.9 (Python Software Foundation, Wilmington, DE, USA) and relevant statistical and machine learning libraries, including NumPy version 1.26.4 (NumPy Developers, Cheyenne, WY, USA), pandas version 2.2.2 (pandas Development Team, Dallas, TX, USA), scikit-learn version 1.5.2 (scikit-learn Developers, Paris, France), matplotlib version 3.9.2 (matplotlib Development Team, USA), and TensorFlow version 2.17.0 (Google LLC, Mountain View, CA, USA).

### 2.7. Statistical Analyses in the Original Cohort

Continuous variables were summarized as the mean with standard deviation (SD) or median with interquartile range (IQR), depending on their distribution, which was assessed using the Shapiro–Wilk test. For group comparisons (DOCAs vs. VKAs), normally distributed continuous variables were analyzed using Student’s *t*-test, while non-normally distributed variables were compared using the Mann–Whitney U test. Categorical variables were presented as absolute frequencies and percentages and compared using the chi-square test or Fisher’s exact test, as appropriate; the latter was applied when expected cell counts were below five. For analytic purposes, patients with single- and double-positive aPL profiles were grouped together as non–triple-positive to improve statistical power and reflect common clinical stratification. Statistical significance was set at a *p*-value < 0.05. All statistical analyses were performed using SPSS software, version 29.0 (IBM Corp., Armonk, NY, USA), and R software, version 4.3.2 (R Foundation for Statistical Computing, Vienna, Austria; www.r-project.org, accessed on 9 February 2025).

## 3. Results

### 3.1. Characteristics and Outcomes in Real-World Patients

Eighty-nine thrombotic APS patients were included: 70 (78.7%) received VKAs, and 19 (21.3%) received DOACs. Among those on DOACs, five received dabigatran, five apixaban, five edoxaban, and four rivaroxaban. Serological profiles were as follows: 34 patients (38.2%) were single-positive, 39 (43.8%) were double-positive, and 16 (18.0%) were triple-positive. LA was the most frequent biomarker (60.7%), following aCL IgG (42.7%). A total of 10/16 triple-positive patients (62.5%) were treated with VKAs.

Baseline characteristics are shown in Table 2. Forty-one patients (46.1%) were female, with a mean age of 56.2 years (±15.6). The median duration of follow-up was 46 (IQR: 36.1–58.7) months. The median time in therapeutic range (TTR) for VKA patients was 63.3% (IQR 52.1–74.5). Significant differences were noted between the groups in sex, dyslipidemia, history of cerebrovascular disease, and serological profile. Female sex was more frequent in the VKA group compared to the DOAC group (52.9% and 21.1%, respectively; *p* = 0.014). Regarding the aPL profile, double positivity was more frequent among patients receiving VKAs (48.6% vs. 26.3%; *p* = 0.032). A total of seven patients (7.9%) died during follow-up, none of them due to thrombotic recurrence or bleeding complications.

During follow-up, thrombotic recurrences occurred in 22 patients (24.7%), all of which represented the first recurrent thrombosis after the index event. These events were symptomatic and confirmed by objective imaging. The mean time to recurrence from the initiation of anticoagulation therapy was 21.8 (±11.4) months. There were 17 recurrences in the VKA group (24.3%), including 11 VTE events, 4 strokes, 1 myocardial infarction, and 1 placental thrombosis. The placental thrombosis was diagnosed by abdominopelvic computed tomography. In the DOAC group, five patients (26.3%) experienced recurrences, including four VTE events and one myocardial infarction; no strokes were observed. Two recurrences occurred with rivaroxaban, two with apixaban, and one with edoxaban. Among patients with recurrence, 4/17 (23.5%) were triple-positive for aPL in the VKA group, and 4/5 (80%) in the DOAC group. Major bleeding occurred in three patients (4.3%) in the VKA group, while no episode of major bleeding was recorded in the DOAC group.

The OR for recurrent thrombosis (arterial or venous) in patients treated with DOACs compared to VKAs was 0.90 (95% CI: 0.28–2.86). For arterial recurrence, the OR was 1.69 (95% CI: 0.19–14.94), and for venous recurrence, 0.70 (95% CI: 0.19–2.51). The OR for major bleeding events was 1.70 (95% CI: 0.08–35.46). Table 3 compares thrombotic recurrence by triple- and non-triple-positive patients. For triple positivity, the OR for overall recurrence was 0.67 (95% CI: 0.10–4.35); for arterial recurrence, 1.75 (95% CI: 0.13–23.70), and for venous recurrence, 0.42 (95% CI: 0.05–3.43). Among non-triple-positive patients, the OR for overall recurrence was 2.77 (95% CI: 0.32–23.64); for arterial recurrence, 1.57 (95% CI: 0.08–31.89), and for venous recurrence, 1.76 (95% CI: 0.20–15.52). Additionally, in patients with index arterial thrombosis, recurrence occurred in 38.9% of those receiving VKAs (7/18) and 50.0% of those receiving DOACs (4/8). The OR for recurrence with DOACs compared to VKAs was 1.57 (95% CI: 0.29–8.42; *p* = 0.683). Among patients with index venous thrombosis, recurrence occurred in 19.2% of those receiving VKAs (10/52) and 9.1% of those receiving DOACs (1/11). The odds ratio for recurrence with DOACs compared to VKAs was 0.42 (95% CI: 0.05–3.67; *p* = 0.671).

### 3.2. Digital Twin

#### 3.2.1. Non-Conditioned Digital Twin Cohort and Internal Structure Validation

**Figure 2:**
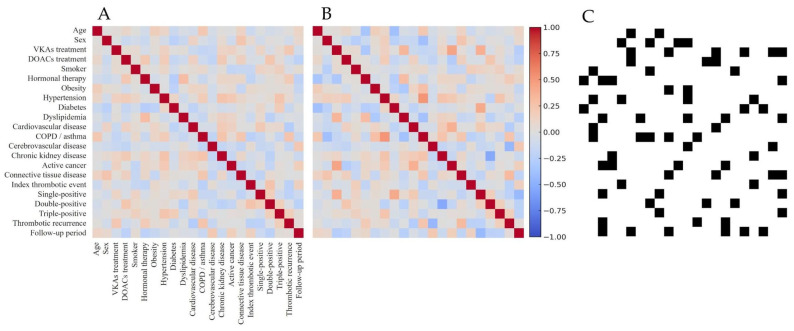
Spearman correlation matrices of encoded clinical variables. (**A**) shows the real-world cohort, and (**B**) shows the CGAN-generated digital twin cohort. (**C**) highlights in black the pairwise correlations that differ by more than 0.1 in absolute value, illustrating high structural similarity between both datasets.

An initial non-conditioned DT cohort was generated using GANs trained on the real-world APS dataset. Ten independent DT cohorts were created using the same CGAN configuration and training parameters. Bootstrap resampling and variable harmonization were applied to preserve the distribution of key variables, including age, sex, index thrombosis type, aPL profile, follow-up period, treatment groups, and recurrence. Across all synthetic cohorts, ASMDs for individual variables comparing synthetic and real-world data were <0.1. The overall MASMD was 0.073 (±0.041). Continuous variables were winsorized at the 2.5th and 97.5th percentiles before training; this preprocessing step did not alter the distributions of key variables or affect validation metrics. All baseline covariates demonstrated adequate balance in the internal comparison of DOAC and VKA subgroups within the non-conditioned DT. ASMDs were 0.063 for age, 0.048 for sex, 0.071 for index thrombotic event, 0.059 for serological phenotype, and 0.034 for follow-up duration. The overall MASMD was 0.060 (±0.023), well below the conventional 0.1 threshold for covariate balance. Spearman correlation matrices were computed for both real and synthetic datasets. Among all pairwise comparisons, 85.4% of correlations in the synthetic cohort differed by less than 0.1 in absolute value relative to the real data. This concordance demonstrated the preservation of the internal dependency structure between variables (Figure 2).

#### 3.2.2. Conditioned Twins and Treatment Effect Estimation

A conditioned DT twin cohort was constructed using CGAN-generated data and 1:1 propensity score matching based on age, sex, index thrombosis type, follow-up duration, and aPL profile. After matching, covariate balance between the DOAC and VKA subgroups was achieved, with ASMDs of 0.026 for age, 0.028 for sex, 0.071 for index thrombosis type, 0.059 for follow-up duration, and 0.088 for aPL phenotype. The overall MASMD was 0.054 (±0.028). The conditioned cohort had a mean age of 55 (±14) years, with 43% women and 17% triple-positive aPL. A logistic regression model applied to the conditioned DT cohort estimated a recurrence rate of 19.9% if all individuals were treated with VKAs and 24.2% if treated with DOACs, yielding an absolute difference of 4.3%. Robustness of the generative model was assessed by creating 10 independent non-conditioned digital twin cohorts. MASMD values compared to the original dataset ranged from 0.048 to 0.102 (mean of 0.069 ± 0.019).

#### 3.2.3. Sensitivity Analysis

**Figure 3:**
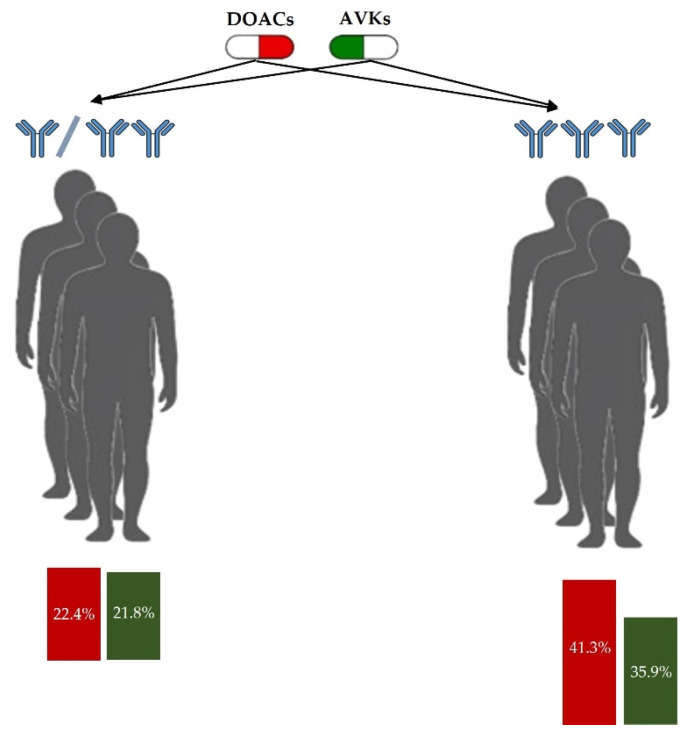
Predicted thrombotic recurrence risk in the digital twin cohort according to antiphospholipid antibody profile. The left panel shows non-triple-positive patients, while the right panel shows triple-positive patients.

A sensitivity analysis was performed by classifying patients into triple-positive and non-triple-positive groups. Recurrence risk increased with aPL burden in the triple-positive subgroup (Figure 3). Among triple-positive patients, DOAC treatment was associated with a predicted recurrence probability of 41.3%, compared to 35.9% with VKAs, yielding an absolute difference of +5.4%. In contrast, among non–triple-positive patients, the difference between treatment groups was minimal (22.4% for DOACs vs. 21.8% for VKAs; +0.6%).

Another sensitivity analysis was also performed, simulating that all patients had presented with an index arterial or venous thrombosis. Under the arterial scenario, predicted recurrence risk was markedly higher in patients receiving DOACs than those on VKAs, with an absolute difference of +21.7%. In contrast, recurrence rates were comparable between treatment groups when assuming an index venous thrombosis for all patients, with a negligible difference of −0.5% (Table 4).

## 4. Discussion

To our knowledge, this is the first application of digital twin technology in thrombosis, specifically addressing treatment strategies in APS. We used this approach to evaluate the comparative effectiveness of DOACs vs. VKAs in this clinical setting.

DTs are dynamic digital replicas of physical entities, processes, or systems that mirror real-world behavior. This technology originated in the aerospace sector. In the 1960s, NASA pioneered the examination of physical objects using DTs. This approach was employed during the Apollo 13 mission [19]. NASA used multiple simulators to evaluate the failure and extended a physical spacecraft model to include digital components. These DTs were the first of their kind, allowing for continuous data ingestion to model the events leading up to the accident, enabling forensic analysis and the exploration of the next steps. Over the last two decades, DT technology has been employed to simulate flights, monitor aircraft health, diagnose structural damage, optimize operational parameters, and enhance system reliability [20]. In the industrial arena, DTs have enabled real-time monitoring, predictive maintenance, and process optimization, leading to marked improvements in operational efficiency and cost reduction [21,22,23].

Healthcare applications of DTs offer the potential to simulate complex patient scenarios, support predictive diagnostics, and enable real-time, personalized treatment planning based on real-world data. In recent years, several systematic reviews have explored the implementation of DTs across diverse medical fields. John et al. study focused on DTs in prostate cancer care, illustrating their ability to simulate disease trajectories and optimize treatment responses [24]. Another meta-analysis reviewed the integration of 3D and 4D digital human modeling with extended reality (XR) technologies in neurorehabilitation, highlighting improved clinical outcomes through personalized virtual representations [25]. In radiology, Faiella et al. discussed the integration of multimodal imaging, artificial intelligence, and computational modeling to construct dynamic, patient-specific DTs [26].

One of the key advantages of DTs in healthcare lies in their ability to perform counterfactual analyses in underrepresented patient populations and rare clinical scenarios, where small sample sizes and clinical heterogeneity often limit the generalizability of RCTs. Thrombotic APS provides an illustrative example. Current evidence from RCTs comparing DOACs and VKAs remains inconclusive. Some of these trials were prematurely interrupted due to safety concerns or recruitment challenges, limiting their power to draw definitive conclusions on the role of DOACs. In our study, the DT approach enabled the generation of synthetic cohorts with optimal covariate balance across key clinical variables. This strategy allowed for the simulation of challenging scenarios, such as a triple-positive population, or a cohort composed exclusively of patients with index arterial thromboses that are rarely feasible to isolate in observational datasets. In the DT cohort, recurrence rates were notably higher among patients with triple-positive serology and arterial index events treated with DOACs. In contrast, recurrence rates were similar between treatment arms in patients with venous index events and non-triple-positive profiles. These observations are in line with patterns reported in the clinical trials [6,7,8,9] and several observational studies [27]. However, the DT approach enabled structured simulation of these clinical scenarios, overcoming the small sample sizes and heterogeneity.

In our real-world cohort, four venous thrombotic recurrences were reported among patients treated with DOACs. Although the absolute number of events is small, the percentage is relatively high, partly due to the limited sample size, which implies that each event carries greater statistical weight. This rate is higher than that reported in previous observational studies, which found venous recurrence rates ranging from 5% to 8% in DOAC-treated APS patients [28]. Notably, those studies included a lower proportion of triple aPL positivity patients compared to our cohort, which may further explain the elevated recurrence rate observed in our series.

This study has several limitations. First, it was based on observational data with a modest sample size, particularly in the DOAC subgroup, which may limit the statistical power and the robustness of subgroup analyses. However, DT technology partially mitigates this limitation by enabling data harmonization, synthetic population expansion, and simulation of complex scenarios. Second, although the generative models preserved key multivariable structures and demonstrated acceptable internal validity, synthetic data inevitably incorporate a degree of statistical noise. This limitation may result in slight discrepancies with real-world estimates. However, such variability is expected and desirable in DT design, as perfect replication would imply overfitting and reduce generalizability. Third, the DAG integrated into the CGAN architecture was constructed based on clinical knowledge rather than data-driven inference. This could lead to bias through misclassification or omission of relevant causal pathways. Residual confounding from unmeasured variables cannot be entirely excluded. Fourth, the grouping of single- and double-positive patients into a single non-triple-positive category was driven by the limited sample size. Although this decision may introduce some heterogeneity, it is supported by existing evidence showing that the recurrent thrombotic risk is similar in patients with single and double positivity. Lastly, several clinical variables, such as INR values or aPL titers, were excluded from the DAG due to incomplete data availability or incompatibility across treatment groups. For instance, TTR does not apply to those receiving DOACs.

## 5. Conclusions

In conclusion, DT modeling offers a robust and innovative approach to simulate thrombotic recurrence risk in APS patients, especially in understudied subpopulations. The consistency between DT-derived outcomes and real-world clinical data supports its potential utility in precision medicine and individualized decision-making.

## Figures and Tables

**Table 1:** Overview of clinical trials assessing the efficacy and safety of direct oral anticoagulants for secondary prevention of thrombotic antiphospholipid syndrome.

	RAPS [6]	TRAPS [7]	Ordi-Ros et al. [8]	ASTRO-APS [9]
Year	2016	2018	2019	2022
Country	United Kingdom	Italy	Spain	United States
Index thrombotic event	VTE	Arterial and venous	Arterial and venous	Arterial and venous
Median follow-up in months	7	20.4	36	12
DOAC/comparison	Rivaroxaban 20 mg day vs. warfarin (INR 2.5)	Rivaroxaban 20 or 15 mg day vs. warfarin (INR 2.5)	Rivaroxaban 20 or 15 mg day vs. VKA (INR 2–3)	Apixaban 5 or 2.5 mg twice daily vs. warfarin (INR 2–3)
Sample size	57/59	59/61	95/95	23/25
Mean age	47/50	46.5/46.1	47/51 *	46/48.5
Female sex, %	74/71	66/62	64/63	83/84
aPL profile, %				
Simple	60/48	0/0	34/32	22/30
Double	28/32	0/0	5/8	17/8
Triple	12/20	100/100	61/60	30/28
Recurrent thrombosis				
Total	None for both groups	7 (12%) vs. 0; *p* = 0.005	11 (11.6%) vs. 3 (6.3%); *p* = 0.21	6 (26%) vs. 0; *p* = 0.008
Arterial	―	7 (12%) ^†^ vs. 0	10 (10.5%) ^§^ vs. 3 (3.2%); *p* = 0.06	6 (26%) ^€^ vs. 0; *p* = 0.008
Venous	―	0 vs. 0	2 (2.1%) vs. 3 (3.2%); *p* = 0.65	0 vs. 0
Major bleeding	None for both groups	4 (7%) vs. 2 (3%); *p* = 0.30	6 (6.3%) vs. 7 (7.4%); *p* = 0.77	0 vs. 1 (4%), *p* = 1.0

* Ordi-Ros et al. used median and IQR for age; ^†^ 4 strokes and 3 myocardial infarctions; ^§^ 9 of the 10 arterial events were strokes (authors did not specify the aPL profile); ^€^ all reported events were strokes. Two occurred in patients with triple positivity, one in a patient with double positivity, and one with single positivity. In two cases, the aPL profile was not specified. Abbreviations: APLs, antiphospholipid antibodies; DOAC, direct oral anticoagulant; INR, International Normalized Ratio; VKA, vitamin K antagonist; VTE, venous thromboembolism.

**Table 2:** Clinical characteristics and outcomes in the real cohort of patients with thrombotic antiphospholipid syndrome.

Baseline Characteristics	Total (n = 89)	VKA Group (n = 70)	DOAC Group (n = 19)	*p*-Value
Demographic data				
Age (mean ± SD)	56.2 (15.6)	55.2 (16.1)	60.1 (12.9)	0.328
Female sex, n (%)	41 (46.1)	37 (52.9)	4 (21.1)	0.014
Previous conditions, n (%)				
Hypertension	44 (49.4)	32 (45.7)	12 (63.1)	0.177
Diabetes	19 (21.3)	12 (17.1)	7 (36.8)	0.063
Dyslipidemia	38 (42.7)	26 (37.1)	12 (63.2)	0.042
Obesity (BMI ≥ 30 kg/m^2^)	32 (36)	24 (34.3)	8 (42.1)	0.529
Smoking	37 (41.6)	27 (38.6)	10 (52.6)	0.270
Chronic heart failure	13 (14.6)	9 (12.8)	4 (21.1)	0.370
COPD/asthma	29 (32.6)	25 (35.7)	4 (21.1)	0.227
Cerebrovascular disease	13 (14.6)	13 (18.6)	0	0.042
Chronic kidney disease	11 (12.4)	10 (14.3)	1 (5.3)	0.289
Connective tissue disease *	12 (13.5)	11 (15.7)	1 (5.3)	0.237
Systemic lupus erythematosus	5 (5.6)	5	0	0.230
Active cancer	5 (5.6)	4 (5.7)	1 (5.3)	0.940
Concomitant antiplatelet therapy	7 (7.9)	5 (7.1)	2 (10.5)	0.638
Index thrombotic event, n (%)				
Arterial	26 (29.2)	18 (25.7)	8 (42.1)	0.267
Venous	63 (70.8)	52 (74.3)	11 (57.9)	0.267
Type of index arterial or venous thrombotic event, n (%)				
Stroke	17 (19.1)	14 (20.0)	3 (15.8)	0.628
Myocardial infarction	5 (5.6)	2 (2.8)	3 (15.8)	0.063
Peripheral artery disease	2 (2.2)	1 (1.4)	1 (5.3)	0.383
Mesenteric artery thrombosis	1 (1.1)	1 (1.4)	0	0.802
Thrombotic endocarditis	1 (1.1)	0	1 (5.3)	0.213
VTE	63 (70.8)	52 (74.3)	11 (57.9)	0.267
Serological profile, n (%)				
Single-positive	34 (38.2)	26 (37.1)	8 (42.1)	0.418
Double-positive	39 (43.8)	34 (48.6)	5 (26.3)	0.032
Triple-positive	16 (18.0)	10 (14.3)	6 (31.6)	0.082
Type of serological profile, n (%)				
Lupus anticoagulant (LA)	54 (60.7)	42 (60.0)	12 (63.1)	0.803
Anticardiolipin antibodies (aCL)	56 (62.9)	42 (60.0)	14 (73.7)	0.273
Anti-beta 2 glycoprotein I antibodies (anti-β2GPI)	52 (58.4)	39 (55.7)	13 (68.4)	0.319
Another thrombophilia, n (%)	4 (4.5)	3 (4.3)	1 (5.3)	0.855
Thrombotic recurrence during follow-up, n (%)				
Total	22 (24.7)	17 (24.3)	5 (26.3)	0.856
Arterial	7 (7.9)	6 (8.5)	1 (5.3)	0.635
Venous	15 (16.8)	11 (15.7)	4 (21.1)	0.730
Type of arterial or venous thrombosis during follow-up, n (%)				
Stroke	4 (4.5)	4 (5.7)	0	0.574
Myocardial infarction	2 (2.2)	1 (1.4)	1 (5.3)	0.383
Placental thrombosis	1 (1.1)	1 (1.4)	0	0.802
VTE	15 (16.8)	11 (15.7)	4 (21.1)	0.730
Mean time to thrombotic recurrence (months)	21.8	23.2	16.6	0.195
Hemoglobin (median, IQR) ^†^	13.5 (12.6–14.8)	13.4 (12.2–14.7)	13.8 (12.8–14.9)	0.350
Platelets (median, IQR) ^†^	203 (160–239)	198 (155–237)	222 (179–246)	0.280
Major bleeding during follow-up, n (%)	3 (3.4)	3 (4.3)	0	0.359
Follow-up period (median, IQR)	46 (36.1–58.7)	48 (38.2–60.5)	43 (30.1–52.0)	0.138
Mortality, n (%)	7 (7.9)	6 (8.5)	1 (5.2)	0.635

* Four cases of Sjögren’s syndrome, one case of rheumatoid arthritis, one case of undifferentiated connective tissue disease, and one case of systemic sclerosis in the VKA group. In the DOAC group, 1 case of Sjögren’s syndrome. ^†^ In patients with thrombotic recurrence at the time of recurrence. Abbreviations: BMI, body mass index; COPD, chronic obstructive pulmonary disease; CVST, cerebral venous sinus thrombosis; DOACs, direct oral anticoagulants; IQR, interquartile range; SD, standard deviation; VKAs, vitamin K antagonists; VTE, venous thromboembolism.

**Table 3:** Logistic regression models for thrombotic recurrence according to aPL profile and treatment group.

aPL Profile	Treatment Comparison	Outcome	OR (95% CI)	*p*-Value
All patients	DOACs vs. VKAs	Total thrombosis	0.90 (0.28–2.86)	0.859
		Arterial thrombosis	1.69 (0.19–14.94)	0.637
		Venous thrombosis	0.70 (0.19–2.51)	0.588
Triple-positive	DOACs vs. VKAs	Total thrombosis	0.67 (0.10–4.35)	0.677
		Arterial thrombosis	1.75 (0.13–23.70)	0.673
		Venous thrombosis	0.42 (0.05–3.43)	0.421
Non-triple-positive	DOACs vs. VKAs	Total thrombosis	2.77 (0.32–23.64)	0.353
		Arterial thrombosis	1.57 (0.08–31.89)	0.768
		Venous thrombosis	1.76 (0.20–15.52)	0.611

Abbreviations: aPL, antiphospholipid antibodies; CI, confidence interval; DOACs, direct oral anticoagulants; NA, not applicable; OR, odds ratio; VKAs, vitamin K antagonists.

**Table 4:** Predicted thrombotic recurrence risk according to index thrombosis in the conditioned digital twin cohort.

Type of Index Thrombosis	VKAs—Predicted Recurrence	DOACs—Predicted Recurrence
Arterial	25.1%	46.8%
Venous	18.3%	17.8%

## Data Availability

The data supporting this study’s findings are available from the corresponding author upon reasonable request. Restrictions apply to the availability of these data, which were used under license for this study.

## Abbreviations

The following abbreviations are used in this manuscript:

DOACs	Direct oral anticoagulants
VKAs	Vitamin K antagonists
APS	Antiphospholipid syndrome
DT	Digital twin
CGANs	Generative adversarial networks
DAG	Directed acyclic graph
ASMD	Absolute standardized mean differences
MASMD	Mean absolute standardized mean differences
aPL	Antiphospholipid antibodies
LA	Lupus anticoagulant
aCL	Anti-cardiolipin antibodies
anti-β2GPI	Anti-beta-2-glycoprotein I antibodies
ACR	American College of Rheumatology
EULAR	European Alliance of Associations for Rheumatology
VTE	Venous thromboembolism
RCTs	Randomized controlled trials
OR	Odds ratio
INR	International normalized ratio
SD	Standard deviation
IQR	Interquartile range
TTR	Time therapeutic range
